# Supplementary material for: Evidence of Au(II) and Au(0) States in Bovine Serum Albumin-Au Nanoclusters Revealed by CW-EPR/LEPR and Peculiarities in HR-TEM/STEM Imaging
Source: Nanomaterials (Basel). 2022 Apr 22;12(9):1425. doi: 10.3390/nano12091425 (PMC9105226; doi:10.3390/nano12091425)
Supplement: Supplementary file 1 [file nanomaterials-12-01425-s001.zip › nanomaterials-1678792-supplementary.pdf]

nanomaterials

Supplementary Materials

Evidence of Au(II) and Au(0) States in Bovine Serum Albumin-Au Nanoclusters Revealed by CW-EPR/LEPR and Peculiarities in HR-TEM/STEM Imaging

Radek Ostruszka [1], Giorgio Zoppellaro [2,*], Ondřej Tomanec [2], Dominik Pinkas [3], Vlada Filimonenko [3] and Karolína Šišková [1,*]

1 Department of Experimental Physics, Faculty of Science, Palacký University, tř. 17.listopadu 12, 77900 Olomouc, Czech Republic; radek.ostruszka@upol.cz
2 Regional Centre of Advanced Technologies and Materials, Faculty of Science, Palacký University, tř. 17.listopadu 12, 77900 Olomouc, Czech Republic; ondrej.tomanec@upol.cz
3 Institute of Molecular Genetics of the Czech Academy of Sciences, Microscopy Centre, Electron Microscopy Core Facility, Vídeňská 1083, 142 20 Prague 4, Czech Republic; dominik.pinkas@img.cas.cz (D.P.); vlada.filimonenko@img.cas.cz (V.F.)
* Correspondence: giorgio.zoppellaro@upol.cz (G.Z.); karolina.siskova@upol.cz (K.Š.)

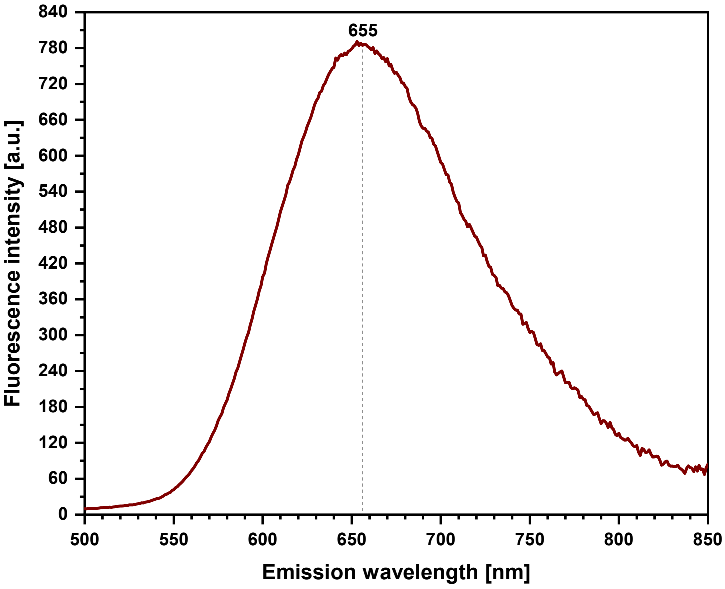

Figure S1. Corrected emission spectrum of AuBSA in deionized water.

Table S1. Quantum yield and wavelength of fluorescence maximum of AuBSA.

Sample	Absorbance	Quantum Yield [%]	Wavelength of Fluorescence Maximum [nm]
AuBSA I	0.0425	6.72	655
AuBSA II	0.0453	6.57	654
AuBSA III	0.0435	6.68	655
Average	0.0438	6.7	655
Standard Deviation	0.0014	0.1	1

Note: Quantum yields were calculated from corrected emission spectra and with respect to DCM dissolved in ethanol. DCM in ethanol was chosen as a standard due to the fact that it reveals a very similar excitation wavelength and fluorescence emission profile as AuBSA.

EPR Data treatments. Simulation of the powder EPR spectrum was performed with the WinEPR SimFonia software (V.1.25, EPR Division, Bruker Instruments, Inc., Billerica, USA) using second-order perturbation theory and spherical integration (grid) of 200 (theta), 200 (phi).

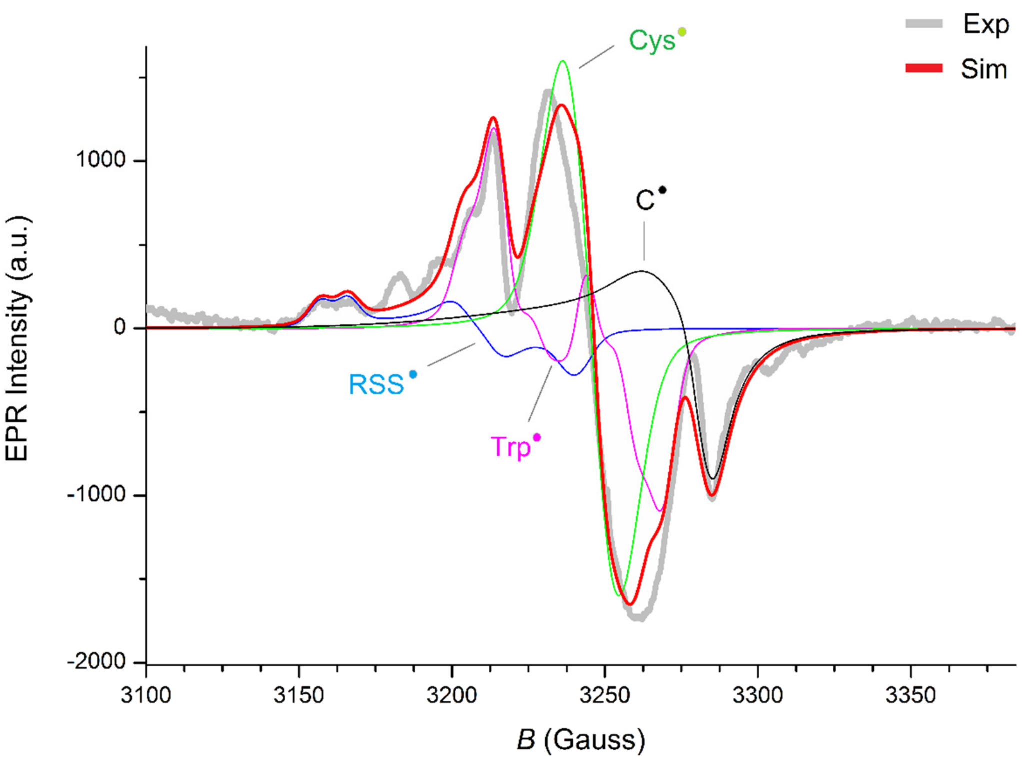

Figure S2. CW-EPR spectrum obtained after 20 min of UV irradiation of BSA in water. Experimental parameters: T = 90 K, 9.0802 GHz frequency, 100kHz modulation frequency, 0.03 s time constant, 1.60 mW applied power, 0.5 mT modulation width, 6 × 100 Gain, 1 min sweep time, and 5 scans accumulated and averaged. The simulation of the various spin components is shown together with their sum (red-line).

Doublet specie, S =1/2, tryptophan radical (Trp•). g_x = 2.002, g_y = 2.003, g_z = 2.003. A_N (xx,yy,zz) = 2.0, G, 2.0 G, 10.0 G; $A_{H\beta 1}$ (xx,yy,zz) = 28.3, G, 28.3 G, 28.3 G; $A_{H\beta 2}$ (xx,yy,zz) = = 13.0, G, 13.0 G, 13.0 G Line-width tensor (x,y,z) = 9.0 G, 9.0 G, 9.0 G. Lorentzian/Gaussian ratio = 0.8. Relative intensity weight = 30.1%

Doublet specie, S =1/2, perthiyl radical (RSS•). g_x = 2.052, g_y = 2.018, g_z = 2.002. Line-width tensor (x,y,z) = 7.0 G, 9.0 G, 9.0 G. Lorentzian/Gaussian ratio = 0.8. Relative intensity weight = 7.1%

Doublet specie, S =1/2, thiyl radical (Cys•). g_x = 2.17, g_y = 2.003, g_z = 2.002. Line-width tensor (x,y,z) = 16.0 G, 16.0 G, 16.0 G. Lorentzian/Gaussian ratio = 0.8. Relative intensity weight = 40.2%

Doublet specie, S =1/2, C-based radical (C•). g_x = 2.012, g_y = 1.984, g_z = 1.977, g_{avg} = 1.991. Line-width tensor (x,y,z) = 60.0 G, 16.0 G, 6.0 G. Lorentzian/Gaussian ratio = 0.8. Relative intensity weight = 22.6%

Preparation of Au-Tyr and Au-Cys: 1 mL solutions of Tyr and/or Cys (12.6 mM and/or 21.1 mM, respectively) were mixed with $HAuCl_4$ (10 mM, 1 mL) under vigorous stirring (600 rpm) after 90 s followed by NaOH addition (1 M, 200 µL), and after another 90 s, microwave heating was applied (10 s, power set to 150 W) as in the case of AuBSA preparation.

UV-vis absorption spectra of Au-Tyr and Au-Cys were then recorded, and the kinetics of potential surface plasmon resonance peak at around 525 nm was followed in the period ranging between 0 and 61.75 mins elapsed from the microwave heating application.

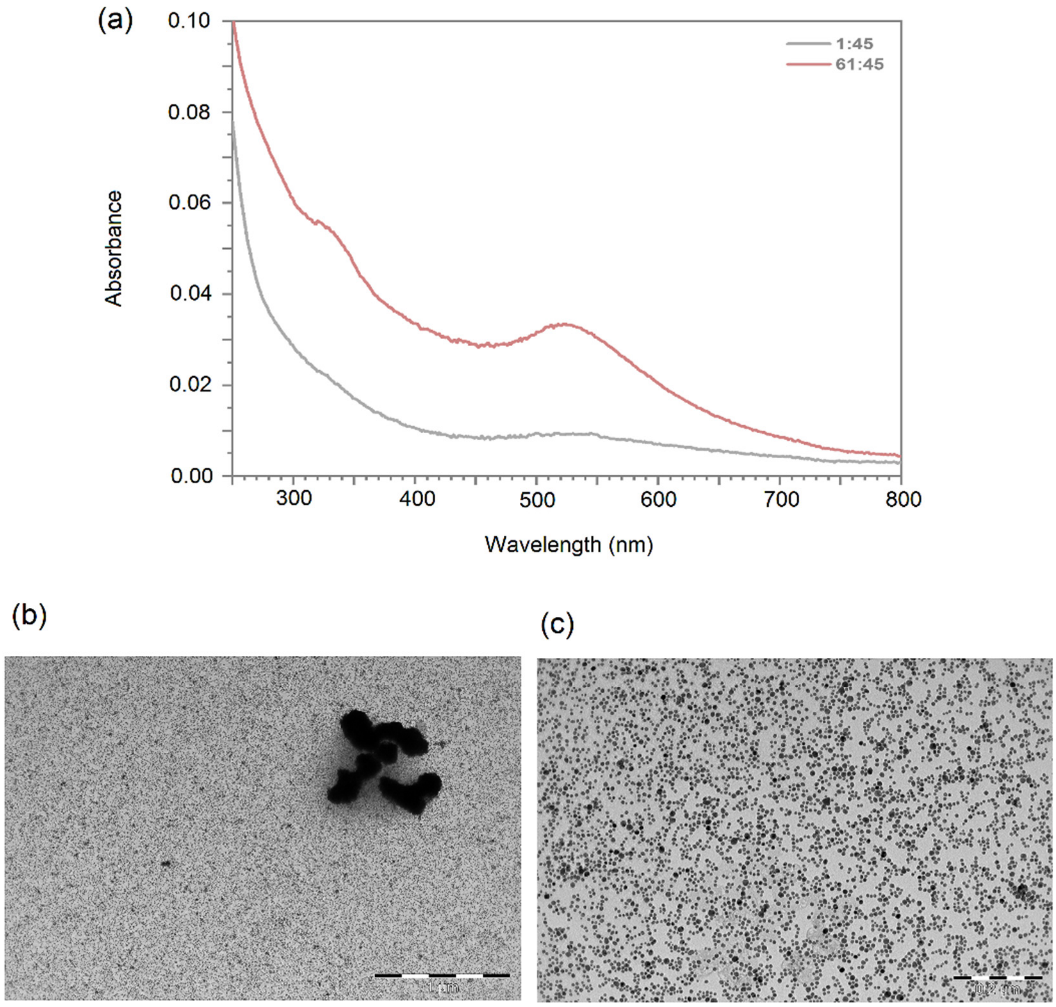

Figure S3. **(a)** Extinction spectra of Au-Tyr in deionized water recorded at 1 minute and 45 seconds after synthesis (grey line) and 61 minutes and 45 seconds after synthesis (red line). Panels (**b**) and (**c**) show the TEM images of Au-Tyr recorded at different magnifications. Scale bar in panel (**b**) equals to 1 µm, and in panel (**c**) equals to 0.2 µm.

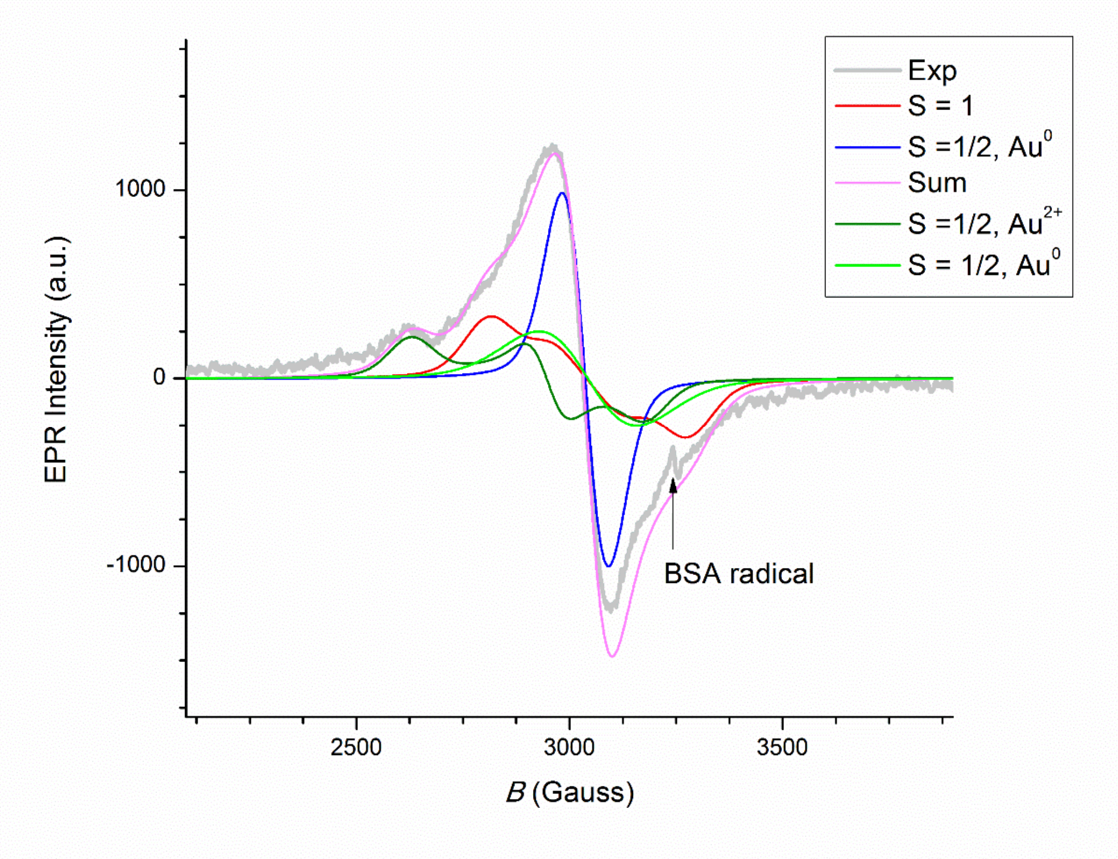

Figure S4. CW-EPR spectrum of AuBSA in water recorded at T = 90 K in dark conditions with the EPR simulation of the diverse spin-components associated to the Au nanoclusters.

Doublet specie, S =1/2, dark green line, Au^{2+}. g_x = 2.47, g_y = 2.20, g_z = 2.04. Line-width tensor (x,y,z) = 100 G, 80 G, 100 G. Lorentzian/Gaussian ratio = 0.63. Relative intensity weight = 12.8%

Doublet specie, S =1/2, blue line, Au^0. g_x = 2.13, g_y = 2.13, g_z = 2.13. Line-width tensor (x,y,z) = 180 G, 100 G, 100 G. Lorentzian/Gaussian ratio = 0.63. Relative intensity weight = 55.2% and S =1/2, green line, Au^0. g_x = 2.13, g_y = 2.13, g_z = 2.13. Line-width tensor (x,y,z) = 230 G, 230 G, 230 G. Lorentzian/Gaussian ratio = 0.63. Relative intensity weight = 13.8%

Triplet specie, S =1, red line. g_x = 2.13, g_y = 2.13, g_z = 2.13. Line-width tensor (x,y,z) = 100 G, 100 G, 100 G. D = 205 G, E = 60 G. Lorentzian/Gaussian ratio = 0.63. Relative intensity weight = 18.2%

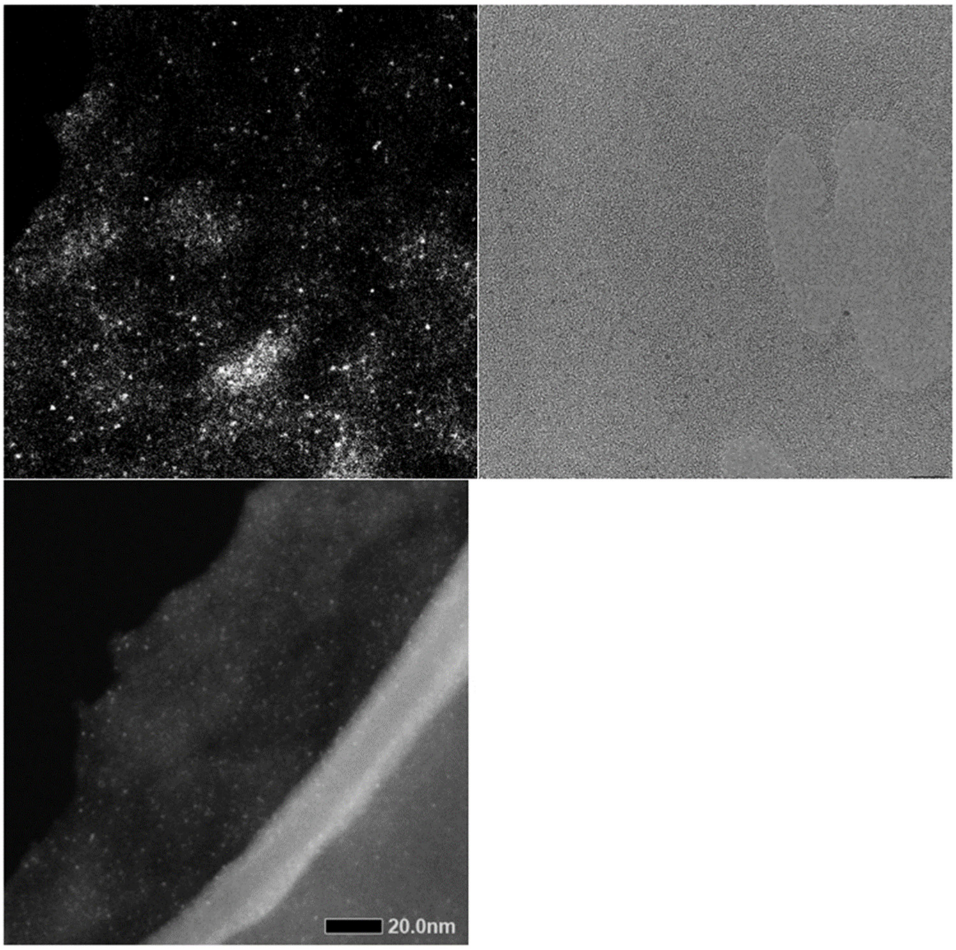

Figure S5. Further STEM images of AuBSA.

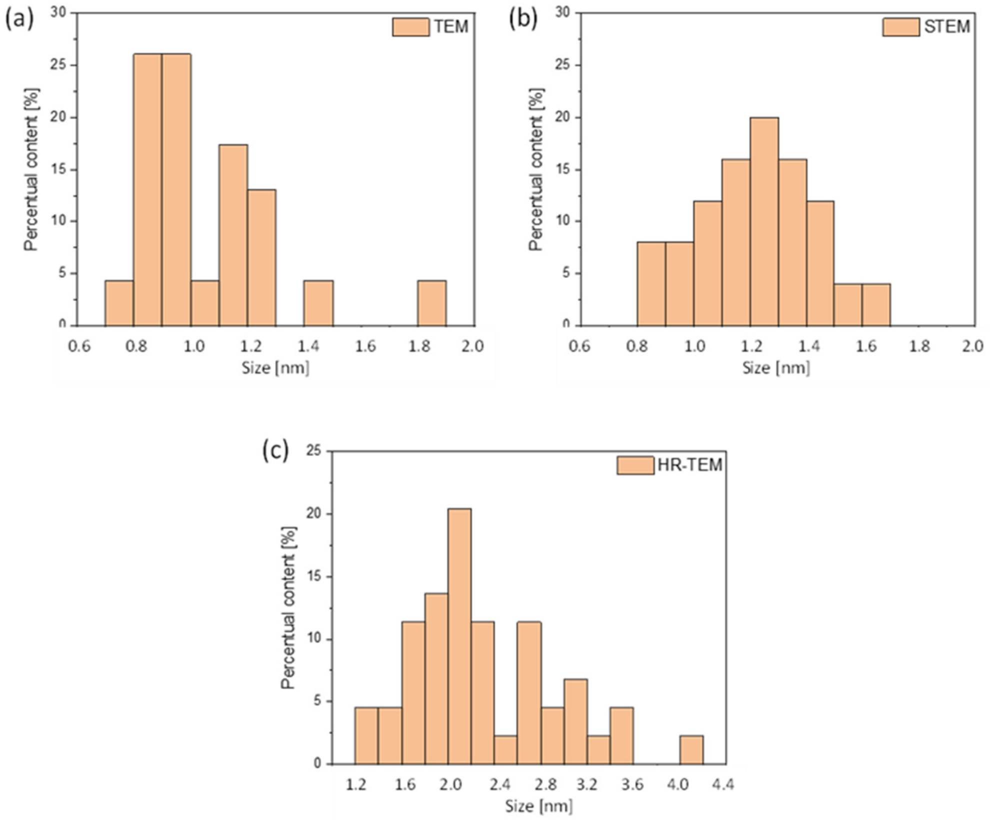

Figure S6. Particle size distributions derived from (**a**) TEM, (**b**) STEM, and/or (**c**) HR-TEM images.

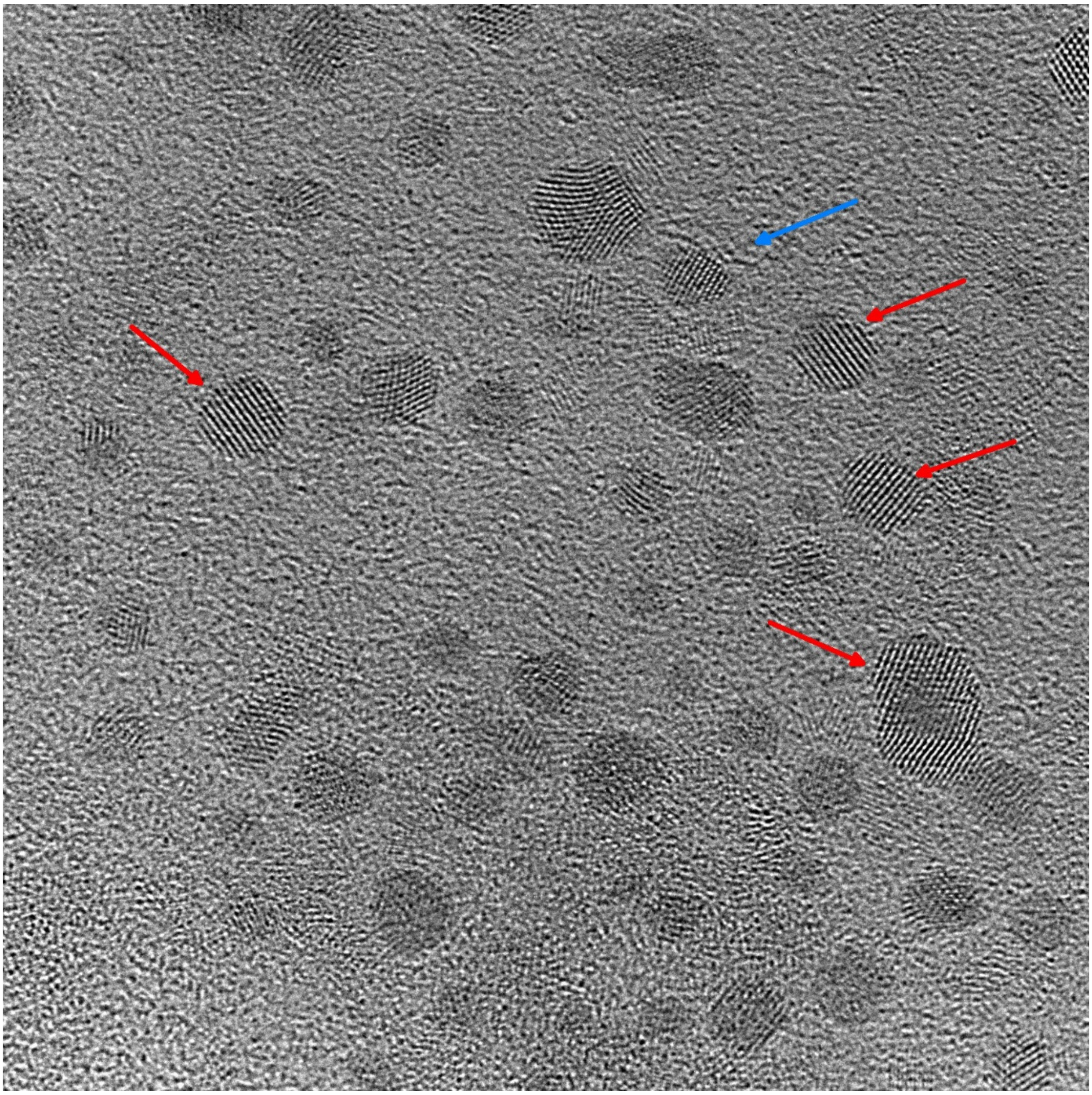

Figure S7. Lattice planes determined for several AuNCs of AuBSA in a particular HR-TEM image. Red arrows indicate Au(111), while the blue arrow represents Au(200).

Filename:	nanomaterials-1678792-supplementary.docx
Directory:	E:\4.22\applsci-1664757
Template:	C:\Users\MDPI\AppData\Roaming\Microsoft\Templates\Normal.dotm
Title:	
Subject:	
Author:	MDPI
Keywords:	
Comments:	
Creation Date:	4/21/2022 9:13:00 PM
Change Number:	3
Last Saved On:	4/21/2022 9:13:00 PM
Last Saved By:	Ivana Kalajdžić
Total Editing Time:	1 Minute
Last Printed On:	4/22/2022 4:52:00 PM
As of Last Complete Printing	
Number of Pages:	7
Number of Words:	910 (approx.)
Number of Characters:	4,627 (approx.)
